# Supplementary material for: Identifying robust predictors of treatment response in trauma-affected refugees: Results from a randomised controlled trial
Source: PLoS One. 2025 Sep 26;20(9):e0324935. doi: 10.1371/journal.pone.0324935 (PMC12469218; doi:10.1371/journal.pone.0324935)
Supplement: S1 Appendix — (DOCX) [file pone.0324935.s001.docx]

**Appendix 1. CTP Predictor index**

|  | | **0** | | **1** | | **2** | | **3** | | **4** | |
| --- | --- | --- | --- | --- | --- | --- | --- | --- | --- | --- | --- |
| **Medical doctor** | | | | | | | | | | | |
| Motivation | | The patient clearly  expresses no  interest in  treatment or feels  compelled to seek  treatment e.g., by  public bodies. | | The patient  expresses great  doubt regarding  treatment efficacy or concerns about  practical issues  such as transport,  and therefore has  limited motivation for starting treatment. | | The clinician  and/or the patient  doubts whether  treatment has  interest. Perhaps  the patient expresses motivation for trying out treatment but doubts that it will  help. | | The patient  expresses a desire  for change and  hope regarding  treatment with  certain reservations, e.g.  a bit of scepticism  about some parts  of the treatment. | | The patient clearly  expresses a desire  for change and  improvement through treatment. The clinician  experiences the  patient as genuinely  interested and as  sincerely believing  that treatment  will help. | |
| Upbringing | | Has throughout  childhood lived  under heavily  disadvantaged  conditions, e.g.  suffering from  war-like  conditions in a  refugee camp,  being orphaned,  hunger, violence  or abuse. | | Has throughout  most of childhood  lived under  stressful conditions but with some reassuring factors, such as having a primary carer most of the time. | | Raised under  relatively safe  conditions but with some deprivation e.g., with a strained  single provider, lack of care/interest from parents, poverty or the like. | | Has throughout  most of childhood  lived under safe  conditions but  suffered slight  deprivation for  shorter periods of  time. | | Has throughout  childhood lived  under fully safe  conditions with  parents, adequate  care and security  as well as finances  to meet material  needs. | |
| Previous relevant  treatment carried out without measurable effect | | Has previously  completed a full  interdisciplinary  treatment  programme with  appropriate  dosage of  antidepressants >  6 months +  psychotherapy > 8 sessions. | | Has previously  completed a full  mono-disciplinary  treatment  programme, e.g.  appropriate  dosage of  antidepressants >  6 months OR  psychotherapy > 8 sessions. | | Treatment has  been partly  attempted e.g.  suboptimal  dosage or few  months of  antidepressants,  and if  psychotherapy  then < 8 sessions. | | Has discussed  mental health  issues with GP but  not received  antidepressants nor  psychotherapy. | | Has never discussed mental health issues previously thus never received  any form of treatment. | |
| Chronic pain | | Has > 2 years  suffered such  severe pain that it  constitutes a  significant  interference with  daily life, preventing the  patient from coping with shopping, cooking, etc. | | Has > 2 years had  constant pain in  many parts of the  body and/or  constant severe  headache, but yet  still cope with  activities of daily  living. | | Is moderately  bothered by pain  but the duration  or scope is less  than 0 and 1. | | Has only limited  pain problems in a  single area of the  body, such as a  knee or hip, and  no significant  reduction of  functional capacity. | | Has no pain or  only transient pain  not exceeding that  of the general  population i.e., a  few days per month with headache. | |
| Chronicity of mental condition | | Current  symptoms, with  the same severity  and impact on  functional  capacity, have  lasted > 10 years. | | Current  symptoms, with  the same severity  and impact on  functional  capacity, have  lasted 2-10 years. | | Current  symptoms, with  the same severity  and impact on  functional  capacity, have  lasted 1-2 years. | | Current  symptoms, with  the same severity  and impact on  functional  capacity, have  lasted < 1 year. | | Current  symptoms, with  the same severity  and impact on  functional  capacity, have  occurred within  the past 6 months. | |
| **Psychologist** | | | | | | | | | | | |
| Understanding of the concept of therapy | | Expresses  absolutely no  understanding of  the concept of  therapy or cannot  see the benefit of  it. | | Expresses limited  understanding of  the concept of  therapy. | | Expresses some  understanding of  the concept of  therapy. | | Expresses good  understanding of  the concept of  therapy. | | Expresses full  understanding of  the concept of  therapy, has  perhaps tried it  previously with  good effect. | |
| Receptiveness/  acceptability to psychological  treatment | | Is by no means  convinced that  psychological  treatment can help or finds it culturally completely unacceptable to see a psychologist. | | Expresses substantial  scepticism about  psychological  treatment, and  strongly doubts  the benefit of the  treatment. | | Is reasonably open towards giving  psychological  treatment a chance, expresses perhaps some scepticism about results. | | Is generally  agreeable towards  starting psychological treatment, has  perhaps heard  good things about  it from others. | | Has had a desire  to see a psychologist, thinks that psychological  treatment is an essential part of the treatment programme and has perhaps had good experiences from previous programmes. | |
| Ability to reflect | | Has no self-knowledge and  reflectivity, completely black  and white simplistic image of oneself and the  surroundings. | | Has rather limited  self-knowledge  and reflectivity. | | Has some self-knowledge but appears to lack a fully realistic view of own situation and resources, which, for example, are seen in an overly positive or negative light. | | Has good self-knowledge and  reflectivity with only few exceptions e.g., in relation to certain individuals or situations. | | Has excellent self-knowledge and demonstrates good abilities to reflect on own situation, resources, and limitations. | |
| Motivation for active participation | | Appears completely passive and entirely dismissive towards actively participating e.g., doing homework. | | Contributes only to a lesser extent actively and appears mostly passive and appellant. | | Expresses an interest in trying to participate actively, but the clinician doubts the patient’s  Actual motivation/ability to contribute actively. | | Expresses a wish to participate actively, but has few reservations e.g., concerns about own abilities to master the given assignments. | | Clearly expresses understanding of the necessity of active participation and willingness to work actively for improvement, both during and between sessions e.g., through homework. | |
| Cognitive resources (memory, concentration, ability to stay focused) | | Has no ability to remember or understand even basic information/need information to be repeated many times, and the patient seems absent and is unable to follow the conversation. | | Memory is clearly impaired, manages only to stay focused for a short while at a time. | | Memory or ability to concentrate is undoubtedly somewhat reduced, but the patient manages to stay focused and follow the conversation most of the time. | | Has only minor difficulty staying focused e.g., towards the end of the session. | | Has no problems with memory or concentration, manages with ease to follow the conversation and asks relevant questions. | |
| **Social counsellor** | | | | | | | | | | | |
| Social relations | | Has none or less than weekly contact with others (friends/family). | | Has practically no friends and barely has sufficient contact with immediate family  (spouse, children). | | Has sufficient contact with immediate family (spouse, children), but sees few people beyond.  that. | | Has sufficient contact with both immediate and more distant family and also has a few friends. | | Has good contact with family (if family is in Denmark), sees several friends, and actively seeks social  social contexts in  day-to-day life. | |
| Daily activity | | No regular weekly occupation/ activity (only comes out for mandatory appointments e.g., with doctor/ municipality). | | Few weekly activities, but no regular activities (for example, comes occasionally to activities e.g., FAKTI). | | A few regular weekly activities (for example, language school once a week, physical activity, volunteer work or similar). | | Some regular daily activities (e.g., attending language school multiple days per week). | | Regular full-time occupation/activity (e.g., work, study, internship or similar) | |
| Job situation | | Uncertain sporadic contact with the job centre as required, without a plan or job. | | Receives cash benefit, mandatory contact with the job center, uncertain plan. | | In clarification process due to illness, such as reduced occupation, flex job or disability pension. Has a plan for activities and treatment. Follows up with job center or unemployment insurance funds. | | Some attachment to the labour market in the form of part-time work or flex job and no attachment to the job centre. Or without contact to the labour market or job centre as the patient for example is a student, on retirement or supported by a spouse. | | Work, full-time occupation | |
| Self-perceived financial situation | | Income is not sufficient to cover daily expenses and must borrow money most months. | | Income is rarely sufficient to cover daily expenses and often must save money at the end of the month. | | Income is just enough to cover daily expenses but not sufficient if there are extra expenses such as additional bills. | | Income is sufficient for regular expenses but must save for things like vacation and leisure activities. | | Income is sufficient, feels financially secure. | |
| Dwelling | | Homeless | | Uncertain housing  situation / only  temporary  accommodation. | | Has dwelling but with obvious  insufficient space  or issues with its  location. | | Seemingly  sufficient housing  conditions in  relation to needs,  but the patient is  still unsatisfied  e.g., with the costs  or size of the  place. | | Lives under fully  satisfying  conditions. | |
| Integration | | Lives as if  completely left  out of the  community, does  not speak Danish,  and has no  contact with  anyone outside  own ethnic group. | | Feels poorly  integrated and  excluded from the  community. | | Has some contact  with the  surrounding  community but  feels in other  ways excluded  e.g., through  language  problems or  negative  experiences with  discrimination or  the like. | | Feels well-integrated,  speaks  Danish well  enough to get by  in everyday life. | | See oneself as a  Dane and speaks  Danish fluently.  Sees Denmark as a  home country. | |
