# Supplementary material for: Identifying robust predictors of treatment response in trauma-affected refugees: Results from a randomised controlled trial
Source: PLoS One. 2025 Sep 26;20(9):e0324935. doi: 10.1371/journal.pone.0324935 (PMC12469218; doi:10.1371/journal.pone.0324935)
Supplement: Attachment — Submitted filename: Comments to Authors.pdf [file pone.0324935.s002.pdf]

Comments to Authors

This replication study robustly confirms several previously reported predictors of refugee treatment response—especially younger age, lower baseline pain, and five “psychotherapy-readiness” items. Analytic methods are sound. I have some comments for the paper.

Major concerns

1. Methods

Multiple Testing: Discuss risk of false positives and use using $p < 0.05$ without adjustment.

2. Results

Direction of Effects: Explicitly state whether each predictor is linked to greater or lesser symptom improvement (e.g., refugee status predicted less PTSD change; 5–10 years impairment predicted poorer depression outcome).

3. Results

Table 2 Accuracy: Show denominators for each percentage so figures match actual N. For example, Migrant status / Refugee / 165 / 75.69. N is 218, not 219.

Minor concerns

1. Correct the typos such as “in-depht” to “in-depth” and “1th of June” to “1st of June” in the Methods. Remove stray commas and semicolons as noted (e.g. “we have previously published seven papers identifying different predictors” rather than “predictors of treatment response, (17, …)” which currently has a comma typo).

2. Since the predictor index involves clinician ratings, consider mentioning whether multiple raters were involved and if any training or consistency checks were in place. In the Discussion, you rightly plan to improve inter-rater reliability in future – that’s good. Even a brief mention of current inter-rater agreement (if known) would be useful for readers to gauge the quality of those measures.

3. Use consistent terminology for the CTP predictor index / psychotherapy-readiness construct. For example, if introducing it as the “CTP predictor index” in Methods, later refer to it simply as “predictor index” or psychotherapy readiness index (if you choose that term) rather than varying the name. Similarly, when referring to change in scores, consider stating “improvement (reduction in symptoms)” to remind the reader that a decrease in HTQ/HAM-D is an improvement.
