# Supplementary material for: Identifying robust predictors of treatment response in trauma-affected refugees: Results from a randomised controlled trial
Source: PLoS One. 2025 Sep 26;20(9):e0324935. doi: 10.1371/journal.pone.0324935 (PMC12469218; doi:10.1371/journal.pone.0324935)
Supplement: Attachment — Submitted filename: Response to Reviewers.docx [file pone.0324935.s004.docx]

**Dear Editor and Reviewers,**

**We would like to thank the reviewers for their thoughtful and constructive comments. We greatly appreciate the time and effort invested in reviewing our manuscript. The feedback and suggestions have been very helpful and have contributed to improving the clarity and overall quality of the paper.**

**Below we address each comment in detail.**

**Journal Requirements:**

When submitting your revision, we need you to address these additional requirements.

1. Please ensure that your manuscript meets PLOS ONE's style requirements, including those for file naming. The PLOS ONE style templates can be found at

<https://journals.plos.org/plosone/s/file?id=wjVg/PLOSOne_formatting_sample_main_body.pdf> and [https://journals.plos.org/plosone/s/file?id=ba62/PLOSOne_formatting_sample_title_authors_affiliations.pdf](https://track.editorialmanager.com/CL0/https:%2F%2Fjournals.plos.org%2Fplosone%2Fs%2Ffile%3Fid=ba62%2FPLOSOne_formatting_sample_title_authors_affiliations.pdf/1/010f01980f0176fd-3000cd56-d06c-4da3-9f25-cb50bb44e51f-000000/JnrE8gNljWdvvA-QIM1FjWFL3QbN6-ervWDUipQ_5ic=219)

***Response:*** *Thank you for the reminder. We have now reviewed and adjusted the manuscript and file naming to ensure full compliance with PLOS ONE’s formatting and style requirements, as outlined in the provided templates.*

2. Thank you for stating in your Funding Statement:

[The RCT was supported by grants from TrygFonden (JC, grant number 120354) and Fonden til Lægevidenskabens Fremme (HS, grant number 16-319).]

The funder did not play a role in the study design, data collection or analysis, decision to publish or preparation of the manuscript.].

Please provide an amended statement that declares *all* the funding or sources of support (whether external or internal to your organization) received during this study, as detailed online in our guide for authors at <http://journals.plos.org/plosone/s/submit-now>.  Please also include the statement “There was no additional external funding received for this study.” in your updated Funding Statement.

Please include your amended Funding Statement within your cover letter. We will change the online submission form on your behalf.

***Response:*** *We have revised the Funding Statement in the cover letter to include all sources of support, and we confirm that the funding paragraph has been removed from the manuscript.*

3. Thank you for stating the following in your manuscript:

[The RCT was supported by grants from TrygFonden and Fonden til Lægevidenskabens Fremme (Foundation for Promotion of Medicine).]

We note that you have provided funding information that is currently declared in your Funding Statement. However, funding information should not appear in the Acknowledgments section or other areas of your manuscript. We will only publish funding information present in the Funding Statement section of the online submission form.

Please remove any funding-related text from the manuscript and let us know how you would like to update your Funding Statement. Currently, your Funding Statement reads as follows:

[The RCT was supported by grants from TrygFonden (JC, grant number 120354) and Fonden til Lægevidenskabens Fremme (HS, grant number 16-319).]

Please include your amended statements within your cover letter; we will change the online submission form on your behalf.

***Response:*** *We have added an amended statement in the cover letter and deleted the funding paragraph in the manuscript.*

4. In the online submission form, you indicated that [The data that support the findings of this study are available upon request from the corresponding author. The data are not publicly available due to privacy and ethical restrictions.].

All PLOS journals now require all data underlying the findings described in their manuscript to be freely available to other researchers, either 1. In a public repository, 2. Within the manuscript itself, or 3. Uploaded as supplementary information.

This policy applies to all data except where public deposition would breach compliance with the protocol approved by your research ethics board. If your data cannot be made publicly available for ethical or legal reasons (e.g., public availability would compromise patient privacy), please explain your reasons on resubmission and your exemption request will be escalated for approval.

***Response:*** *We have added the following to the manuscript, section of availability of data and materials.*

*“Public deposition would breach compliance with the protocol approved by the Ethics Committee of the Capital Region of Denmark and the Danish Data Protection Agency. Furthermore, public availability would compromise patient privacy.”*

5. Your ethics statement should only appear in the Methods section of your manuscript. If your ethics statement is written in any section besides the Methods, please delete it from any other section.

### *Response: We have moved the section “Ethics approval and consent to participate” to the Methods section of the manuscript.*

6. Please include captions for your Supporting Information files at the end of your manuscript, and update any in-text citations to match accordingly. Please see our Supporting Information guidelines for more information: [http://journals.plos.org/plosone/s/supporting-information](http://track.editorialmanager.com/CL0/http:%2F%2Fjournals.plos.org%2Fplosone%2Fs%2Fsupporting-information/1/010f01980f0176fd-3000cd56-d06c-4da3-9f25-cb50bb44e51f-000000/f-p48ux8jHDgwWiEhGZBQ6-xAan6Z5gXsrsLaz7mI9w=219).

If the reviewer comments include a recommendation to cite specific previously published works, please review and evaluate these publications to determine whether they are relevant and should be cited. There is no requirement to cite these works unless the editor has indicated otherwise.

***Response:*** *At the end of the manuscript, we have added captions for “Supporting Information: Appendix 1. Predictor index at the Competence Centre for Transcultural Psychiatry”*

Reviewers' comments:

Reviewer's Responses to Questions

Comments to the Author

1. Is the manuscript technically sound, and do the data support the conclusions?

The manuscript must describe a technically sound piece of scientific research with data that supports the conclusions. Experiments must have been conducted rigorously, with appropriate controls, replication, and sample sizes. The conclusions must be drawn appropriately based on the data presented.

Reviewer #1: Partly

Reviewer #2: Yes

Reviewer #3: Yes

2. Has the statistical analysis been performed appropriately and rigorously?

Reviewer #1: No

Reviewer #2: Yes

Reviewer #3: Yes

3. Have the authors made all data underlying the findings in their manuscript fully available?

The [PLOS Data policy](http://track.editorialmanager.com/CL0/http:%2F%2Fwww.plosone.org%2Fstatic%2Fpolicies.action%23sharing/1/010f01980f0176fd-3000cd56-d06c-4da3-9f25-cb50bb44e51f-000000/pzqWu5b1LyCi2uYaxBtTU4C0GvRdDqLIyw1ixyAEVq8=219) requires authors to make all data underlying the findings described in their manuscript fully available without restriction, with rare exception (please refer to the Data Availability Statement in the manuscript PDF file). The data should be provided as part of the manuscript or its supporting information, or deposited to a public repository. For example, in addition to summary statistics, the data points behind means, medians and variance measures should be available. If there are restrictions on publicly sharing data—e.g. participant privacy or use of data from a third party—those must be specified.

Reviewer #1: Yes

Reviewer #2: Yes

Reviewer #3: Yes

4. Is the manuscript presented in an intelligible fashion and written in standard English?

PLOS ONE does not copyedit accepted manuscripts, so the language in submitted articles must be clear, correct, and unambiguous. Any typographical or grammatical errors should be corrected at revision, so please note any specific errors here.

Reviewer #1: Yes

Reviewer #2: No

Reviewer #3: Yes

5. Review Comments to the Author

**Reviewer #1:**

This manuscript presents data analysis from a randomized control trial (RCT) that aims to identify robust predictors of treatment responses, specifically, posttreatment score differences in PTSD symptoms, among trauma-affected refugees in Denmark. The topic is of paramount importance in global public health, given the global burden of refugees. The study was registered as a RCT (with a valid NCT number), and was approved by the respective IRB/Ethics Committee. Some other (statistical) comments were also provided.

1. Methods:

Methods reporting need some work. In the paper, the authors pointed to the reader to look into previous published work on the proposed RCT. However, to maintain the continuity of this current work, some additional effort is needed to briefly reiterate some of the design aspects of the study published in a previous work within this paper.

Specific comments:

(a) For instance, clearly mention the groups that subjects were randomized into. Also, the randomization and allocation concealment should be made very clear (they are NOT the same thing); the trial staff recruiting patients should NOT have the randomization list. Randomization should be prepared by the trial statistician, and he/she would not participate in the recruiting.

***Response***: *thank you for emphasizing the need for more information on the original RCT. We have added some additional information to the manuscript. However, we have not provided the full information on randomization in the current version. If the reviewer/editor finds this necessary, we are happy to add the information.*

*The following has been added to the method section:*

### “*Study design*

*Data for the current study derived from a a randomized controlled superiority trial with an allocation ratio of 1:1:1:1 and an allocation sequence with block size unknown to the investigator.* *The randomization was stratified by gender.*

*The patients were randomised into four groups. All four groups received treatment as usual (TAU), consisting of an interdisciplinary treatment approach, covering a period of 8-12 months, with medicine according to standard at CTP (best clinical practice in the field), physiotherapy, psychoeducation (including sleep hygiene education and relaxation techniques), and manual-based CBT. One group received solely TAU, serving as a control group; the three remaining groups were active treatment groups receiving add-on treatment with either mianserin, IRT, or a combination of both. “*

*We have inserted the in-depth description from the trial protocol below, please see reference 41 and 42):*

*“The actual randomisation procedure is carried out as follows: the randomisation sequence was computer generated by the Department of Biostatistics at the University of Copenhagen with allocation sequence with block size unknown to the investigator. This department is not otherwise involved in the research project. The Department of Biostatistics at the University of Copenhagen drew up a randomisation list. A person, who was neither associated with the treatment nor the management of the trial converted the randomisation list into sealed, sequentially numbered envelopes. The randomisation list is kept at the Department of Biostatistics.*

*Two secretaries not involved in the daily work at CTP manage the envelopes.*

*When a patient is found eligible for inclusion and has signed informed consent, the physician contacts the secretary mentioned above. The secretary opens the envelope and informs the physician about the assigned intervention group. The physician immediately notes the assigned intervention group on the patient record. The secretary notes the date of birth of the randomised patient on a randomisation list in order to enable backtracking of the randomisation process. In the following session, the physician informs the patient about the assigned intervention.*

*The randomization was stratified by gender.”*

(b) Some details on the randomization is needed, which might be already available in the published work. I would advise the authors to put some sentences in that regard with brevity.

***Response***: *Please see response 1a.*

(c) Sample size: The sample size/power statement should also be reiterated, with specific reference to the primary outcome measure (posttreatment score differences).

***Response****: Thank you for the comment. We have added the following paragraph to the manuscript:*

*“The primary outcome in the RCT was the Pittsburgh Sleep Quality Index. Based on previous studies, a minimal clinically important difference of 2.5 points and a standard deviation of 3 were assumed. With 90% power and alpha = 0.05, the required sample size was 128. To account for an expected dropout of 25%, the target sample was increased to 228 participants.”*

(d) Statistical Analysis Plan:

(d1) A major part of the analysis focuses on regression techniques, which are valid under strong Gaussian assumptions of the errors. Did the authors confirm that Gaussian assumptions when fitting the multiple regression with the response (score differences) were valid? If not, they need to seek alternative methods.

***Response:*** *Thank you for the helpful comment regarding regression assumptions. The following paragraph was already included in the Statistics section of the manuscript:*

*“To assess assumptions of linearity, a scatterplot of the included continuous variables was plotted with a superimposed regression line. Visual inspection of these plots revealed a linear relationship between the variables and no outliers of importance. For the variable age, the relationship with treatment response was nonlinear for several dependent variables; thus, both linear and quadratic terms for age were included.”*

*We have now added the following paragraph to address the remaining regression assumptions more explicitly:*

*“Assumptions of independence of errors, homoscedasticity, and normality of residuals were met to a reasonable degree based on visual inspection of residual-versus-fitted plots and Q-Q plots, as well as rvfplot commands in Stata. Multicollinearity was assessed using variance inflation factors (VIF), and no problematic levels were observed.”*

(d2) What's the plan for handling missing values in the design?

***Response:*** *Thank you for this question. Missing posttreatment outcome data were handled using Full Information Maximum Likelihood (FIML) estimation implemented via STATA’s SEM procedure. FIML allows inclusion of all available data under the assumption that data are missing at random, and is considered a robust and appropriate method for handling missingness in longitudinal designs. This approach enabled us to retain participants with incomplete posttreatment data, thereby maximizing statistical power and reducing bias.*

*Please see the last paragraph in the statistics section.*


2. Results & Conclusions:

(a) The authors should check that any statement of significance should be followed by a p-value in the entire Results section. Otherwise, the Results section look OK; it's pretty straightforward.

***Response:*** *Thank you for this comment. We have carefully reviewed the Results section and ensured that all statements of statistical significance are now followed by the corresponding p-values. This has been corrected throughout the Results section for clarity and transparency.*

(b) Conclusions should state that the current findings are ONLY based on the random samples derived from the refugees entering Denmark. For a comprehensive yet robust evaluation of the predictors, they should allude to future studies involving multiple regions/countries, with much larger sample sizes.

### *Response: Thank you for this suggestion. We agree that future research should aim to validate findings across different contexts and populations. We have now added the following sentence to the conclusion:*

### *“To ensure a more comprehensive and robust evaluation of these predictors, future studies should be conducted across multiple regions and countries, using larger and more diverse samples.”*

**Reviewer #2:**

This replication study robustly confirms several previously reported predictors of refugee treatment response—especially younger age, lower baseline pain, and five “psychotherapy-readiness” items. Analytic methods are sound. I have some comments for the paper.


Major concerns
1. Methods
Multiple Testing: Discuss risk of false positives and use using p < 0.05 without adjustment.

***Response:*** *Thank you for this important point. The aim of the current study was to examine whether previously identified treatment outcome predictors from earlier CTP studies could be replicated in a new sample. Thus, the tested predictors were theory-driven and based on prior empirical findings rather than selected through data-driven exploration. Given this predefined and confirmatory approach, we did not apply formal corrections for multiple testing. However, we acknowledge the general risk of inflated Type I error when conducting multiple analyses, and have added a note to the limitations section to reflect this consideration. We have added the following to the limitations section:*

*“While all tested predictors were theory-driven and based on previous findings, multiple comparisons increase the risk of false positives. Although no formal adjustment was applied, findings should be interpreted with caution, particularly where p-values approach the conventional significance threshold.”*

2. Results
Direction of Effects: Explicitly state whether each predictor is linked to greater or lesser symptom improvement (e.g., refugee status predicted less PTSD change; 5–10 years impairment predicted poorer depression outcome).

***Response:*** *Thank you for this important comment. We have clarified the direction of effects for each significant predictor by explicitly stating whether it was associated with greater or lesser symptom improvement. These clarifications have been added in the results section where the predictors for PTSD and depression are described. In addition, we revised the phrasing for consistency and clarity (e.g., “greater symptom improvement,” “poorer treatment outcome”).*

3. Results
Table 2 Accuracy: Show denominators for each percentage so figures match actual N. For example, Migrant status / Refugee / 165 / 75.69. N is 218, not 219.

***Response:*** *Thank you for pointing this out. We have added the denominators for all percentages to ensure accuracy and transparency. The figures now correspond precisely to the actual sample sizes used for each variable (see revised Table 2).*

Minor concerns
1. Correct the typos such as “in-depht” to “in-depth” and “1th of June” to “1st of June” in the Methods. Remove stray commas and semicolons as noted (e.g. “we have previously published seven papers identifying different predictors” rather than “predictors of treatment response, (17, …)” which currently has a comma typo).

***Response:*** *We have corrected the mentioned typos.*

2. Since the predictor index involves clinician ratings, consider mentioning whether multiple raters were involved and if any training or consistency checks were in place. In the Discussion, you rightly plan to improve inter-rater reliability in future – that’s good. Even a brief mention of current inter-rater agreement (if known) would be useful for readers to gauge the quality of those measures.

***Response:*** *Thank you for this comment. Multiple clinicians were involved in rating the predictor variables. All raters received standardized training prior to data collection to ensure a consistent understanding of the rating procedures. However, formal checks of inter-rater reliability were not implemented, and systematic monitoring of consistency between raters was not conducted during the study period.*

*We have added the following to the limitations section: “In the current study, all clinicians received standardized training, but no formal interrater reliability checks were performed, and agreement between raters was not systematically monitored. Future studies should include procedures for evaluating and reporting interrater agreement to strengthen the quality of clinician-rated data.”*

3. Use consistent terminology for the CTP predictor index / psychotherapy-readiness construct. For example, if introducing it as the “CTP predictor index” in Methods, later refer to it simply as “predictor index” or psychotherapy readiness index (if you choose that term) rather than varying the name. Similarly, when referring to change in scores, consider stating “improvement (reduction in symptoms)” to remind the reader that a decrease in HTQ/HAM-D is an improvement.

***Response:*** *Thank you for this suggestion. We have revised the manuscript to ensure consistent use of terminology. The term “CTP predictor index” is now used throughout the manuscript.*

*Furthermore, we have revised the relevant sections in the results to clarify that a reduction in HTQ and HAM-D scores reflects an improvement in symptoms. Specifically, we now refer to “improvement (reduction in symptoms)” when describing change scores, see results section.*

**Reviewer #3**:

Comments: Thank you for giving me an opportunity to review this important study that identifies robust predictors of treatment response in trauma affected refugees based on previous randomized studies. Based on the growing number of refugees with mental health problems, the authors are commedable for coming up with this innovation.
Please see below my minor comments.
Can the authors expand on their recommendations to take care of “ none modifiable risk factors”. In the context of refugee settings and cumulative trauma, personal guilt, maladaptive coping behavior of rumination such as the survivors’ persistent thoughts on how justice or revenge against the perpetrators could be achieved perpetuate various mental health comorbidities.

***Response:*** *Thank you for highlighting this important aspect. We agree that non-modifiable factors such as past trauma exposure and personal guilt may perpetuate mental health problems and are difficult to change directly. Our intention in referring to non-modifiable factors was to emphasize the importance of identifying them early in the clinical process so that they can inform individualized treatment planning. While these factors cannot be altered per se, they can guide clinicians in tailoring interventions (e.g., addressing guilt through trauma-focused therapy, or targeting rumination through metacognitive strategies). We have now added a brief clarification in the Discussion section to reflect this point.*

*“While such factors cannot be changed, they can still inform clinical decision-making; for example, individuals experiencing persistent guilt or rumination may benefit from trauma-focused interventions that address these* *manifestations of distress. “*
